# Eye movement activity in normal human fetuses between 24 and 39 weeks of gestation

**DOI:** 10.1371/journal.pone.0178722

**Published:** 2017-07-12

**Authors:** Hikohiro Okawa, Seiichi Morokuma, Kana Maehara, Akiko Arata, Yoshiyuki Ohmura, Takashi Horinouchi, Yukuo Konishi, Kiyoko Kato

**Affiliations:** 1 Department of Obstetrics and Gynecology, Graduate School of Medical Sciences, Kyushu University, Fukuoka, Japan; 2 Research Center for Environmental and Developmental Medical Sciences, Kyushu University, Fukuoka, Japan; 3 Department of Obstetrics and Gynecology, Kyushu University hospital, Fukuoka, Japan; 4 Division of Physiome, Department of Physiology, Hyogo College of Medicine, Hyogo, Japan; 5 Department of Mechano-Informatics, Graduate School of Information Science and Technology, The University of Tokyo, Tokyo, Japan; 6 Department of Obstetrics and Gynecology, Kurume University School of Medicine, Kurume, Japan; 7 Center for Baby Science, Doshisha University, Kyoto, Japan; Alberta Children's Hospital, CANADA

## Abstract

Rapid eye movement (REM) sleep occurs throughout a relatively large proportion of early development, and normal REM activity appears to be required for healthy brain development. The eye movements (EMs) observed during REM sleep are the most distinctive characteristics of this state. EMs are used as an index of neurological function postnatally, but no specific indices of EM activity exist for fetuses. We aimed to identify and characterize EM activity, particularly EM bursts suggestive of REM periods, in fetuses with a gestational age between 24 and 39 weeks. This cross-sectional study included 84 normal singleton pregnancies. Fetal EMs were monitored using real-time ultrasonography for 60 min and recorded as videos. The videos were manually converted into a time series of EM events, which were then analyzed by piecewise linear regression for various EM characteristics, including EM density, EM burst density, density of EMs in EM bursts, and continuous EM burst time. Two critical points for EM density, EM burst density, and density of EMs in EM bursts were evident at gestation weeks 28–29 and 36–37. Overall EM activity in human fetuses increased until 28–29 weeks of gestation, then again from 36–37 to 38–39 weeks of gestation. These findings may be useful for creating indices of fetal neurological function for prognostic purposes.

## Introduction

Rapid eye movement (REM) sleep occupies a relatively large proportion of early development [1,2]. In rat neonates, for example, pharmacological blockage of REM sleep suppresses brain development (particularly for the cerebral cortex), and such rats demonstrate maladaptive behavior in new environments [3,4]. This and similar studies suggest that normal REM sleep, or at least REM activity, is required for healthy brain development [3–6].

The eye movements (EMs) observed during REM sleep are the most distinctive characteristics of this state. In addition, EMs can be used to assess central nervous system function. For example, in patients with cognitive/intellectual disability, a correlation exists between IQ and EMs, wherein a lower IQ is associated with reduced EMs during REM sleep [7]. Moreover, in patients with infantile epilepsy or autism, the REM number, REM density, and REM bursts are reduced compared to those in normal children [8–13]. In studies of preterm-birth infants born at 32–36 weeks, REM sleep activity was found to correlate with cognitive development when assessed at six months of age, and to provide an indicator of developmental prognosis [14]. In addition, REM sleep activity is used as an index of normal neurological maturation, wherein abnormalities in REM sleep can reflect an underlying neural pathology. However, no specific indices of REM activity exist for fetuses. In addition, it is technically difficult to define REM sleep in a fetus because sleep states are defined largely by their electroencephalographic (EEG) data. EEG cannot be conducted in a fetus in the same way as in adults, making comparisons between developmental states difficult. Thus, while rapid fetal eye movements are potentially representative of REM sleep, it cannot be conclusively demonstrated; therefore, presumed REM activity will instead be described as EM activity.

Bots et al. reported the first use of ultrasonography in the examination of fetal EMs [15]. Fetal EMs can be detected at approximately 14 weeks of gestation, and they not only increase throughout development but also begin to consolidate between 24 and 26 weeks of gestation; the EM frequency increases after 29–30 weeks of gestation [15]. These changes of fetal EM patterns have been supported by studies from Birnholz et al., and the underlying trend in frequency has been supported by studies from Inoue et al. [16–18]; however, no study to date has examined fetal EM patterns in as many subjects or in the amount of detail that we have provided in this study, or evaluated specific indices such as EM density and EM bursts that reflect EM activity during this developmental period.

Investigating EM activity during the fetal period is important for understanding the role of EM activity in neural development, and opens the possibility of developing an index for neurological prognosis and prediction. Thus, the present study aimed to characterize EM activity, including EM density and EM bursts, in fetuses at a gestational age between 24 and 39 weeks.

## Materials and methods

### Fetal population

The study population consisted of 86 normal singleton pregnancies (gestational age 24 to 39 weeks) that received perinatal management at the Maternity and Prenatal Care Unit, Kyushu University Hospital. The present study was cross-sectional and took place between April 2013 and January 2016. None of the selected cases featured fetal abnormalities (i.e., morphological defects, fetal growth restrictions, etc.) or maternal diseases. Gestational age was calculated from the mother’s last menstrual period, and confirmed during the first trimester via serial ultrasonographic measurements of crown-rump length. All of the recruited mothers were non-smokers, with no history of alcohol abuse. During pregnancy, none of the patients received medication, excluding iron and vitamin supplements. The clinical characteristics of the recruited subjects are displayed in Table 1. All patients experienced uneventful deliveries with healthy newborn babies, in which no abnormal neurological findings were identified at 1 month of age. The present study was approved by the institutional review board of Kyushu University (No. 27–51), and informed consent was provided by all mothers prior to the start of the study.

**Table 1:** Characteristics of the 84 fetuses.

Age group (weeks)	n	Gestational age at delivery (weeks.days)	Birth weight (g)	Sex (male/female; n)	Apgar score		pH of the umbilical artery
					1 min	5 min	
24–25	9	39.6 (38.5–40.6)	3136 (2685–3670)	4/5	9 (7–9)	9 (9–10)	7.28 (7.19–7.39)
26–27	10	39.2 (36.3–40.6)	3013 (2340–3330)	3/7	9 (7–9)	9 (9–10)	7.30 (7.26–7.37)
28–29	10	38.5 (36.0–40.1)	3120 (2340–3810)	7/3	8 (7–9)	9 (8–10)	7.28 (7.23–7.35)
30–31	10	39.4 (37.4–42.1)	3138 (2440–3368)	5/5	9 (8–9)	9 (9–10)	7.33 (7.30–7.44)
32–33	11	38.5 (37.0–41.1)	2920 (2565–3155)	7/4	8 (4–9)	9 (7–10)	7.30 (7.18–7.38)
34–35	12	40.0 (38.2–41.1)	3166 (2600–3670)	9/3	9 (7–9)	9 (9–10)	7.26 (7.20–7.35)
36–37	12	39.6 (38.2–41.0)	3479 (3040–3885)	8/4	8 (7–9)	9 (9–10)	7.25 (7.12–7.43)
38–39	10	39.5 (38.4–40.5)	3050 (2765–3590)	6/4	9 (8–9)	9 (8–10)	7.32 (7.18–7.35)
Total	84	39.4 (36.0–42.1)	3080 (2340–3885)	49/35	8 (4–9)	9 (7–10)	7.30 (7.12–7.44)

Median data are shown with ranges.

### Data acquisition

Patients were placed in a semi-recumbent position in a quiet room with relatively low illumination. This position was freely altered when requested by the mother. Observations took place between 13:00–16:00, and at least 2 h after food ingestion. Fetal EMs were monitored using real-time ultrasonography (APLIO 500 TUS-A500; TOSHIBA, Japan, using a PVT-375BT Probe) for 60 min at a frame rate of 30 frames/s or more. Data were recorded in an MP4 format digital video file on a memory card.

### Analytical methods

#### Data processing

We created time series data for the timing of each EM using the video recordings of fetal EMs. For this procedure, the keyboard was tapped in response to the start of every identifiable EM because using frame-by-frame playback to make time series data is labor- and time-intensive. Specially developed software was used to automatically collect indicators relating to EMs (listed below) from these time series data. The time series data were divided into 1-min epochs and set to either an EM period when EM was discerned, or an NEM period when no EM was discerned.

An EM burst has been defined previously [19,20] as EMs with an inter-EM interval of <1 s on two or more occasions. This definition would categorize the majority of burst-type EMs as REMs. An elapsed period of more than 1 s between the last EM and the next during burst formation marked the end of an EM burst [19,20]. We defined the duration for which we could observe the eye movements as “effective observation time”.

The analysis indicators related to EMs were as follows:

1. Effective observation time (min)
  For cases in which more than 80% of the observation time (≥ 48 min) was effective observation time, analyses were performed for the following indicators.
2. EM period rate (%) = EM period (min)/Effective observation time (min) x 100
3. NEM period rate (%) = NEM period (min)/Effective observation time (min) x 100
4. EM density = Total EM number/EM period (min)
5. EM burst density = Total EM burst number/EM period (min)
6. Density of EMs in EM bursts = Number of EMs in EM bursts/EM period (min)
7. EM burst continuous time

#### Fetal developmental groups

Based on the gestational week, cases were divided into eight age-groups of two consecutive weeks each (fetuses at 24–25 weeks, 26–27 weeks, 28–29 weeks, 30–31 weeks, 32–33 weeks, 34–35 weeks, 36–37 weeks, and 38–39 weeks of gestation).

#### Piecewise linear regression analysis

Scattergrams of EM density, EM burst density, density of EMs in EM bursts and continuous EM burst time vs. gestational age were analyzed to identify any ‘critical points’ between 24–25 and 38–39 weeks of gestation using a piecewise linear regression analysis [21,22].

For selecting the best regression equation, Mallows’ *Cp* value is defined by the equation *Cp = RSS/s*^*2*^*-(n-2p)*, where *n* is the number of fetuses, *p* is the number of critical points, *RSS* is the residual sum of the squares from a given combination of *p* points, and *s*^*2*^ is the residual mean square based on the regression, using all points [23,24]. The procedure for selecting the best piecewise linear regression consists of two steps. First, *p* is determined as the smallest *p* for which the *Cp* value is equal to or smaller than *p*. Second, the best combination of critical points is selected as that when the smallest *Cp* value is found among the groupings of critical points [23,24]. In this analytical method, the two extremes of the age range (24–25 and 38–39 gestational weeks) might be detected as critical points, and thus these time points were excluded from the critical points.

The Student’s t-test was used to test differences in the mean values of EM density, EM burst density, continuous EM burst time, and density of EMs in EM bursts at the beginning and end of the gestational age range, as well as at ‘critical points’. All analyses were performed using the statistical software R 3.2.5 (https://www.r-project.org/).

#### Verification of tapping process reliability

To assess the reliability of the tapping process with regard to data recording, a subsequent analysis was performed. Namely, the results obtained by advancing the video frame at 1/30th s were correlated with data obtained by the tapping process. Repeat measurements of the tapping process were performed in three cases. The tapping process was performed independently in each case to calculate both inter-observer (O.H., M.K., and M.S.) and intra-observer (O.H.) intra-class correlation coefficients (ICCs). ICCs were used to assess the reliability of inter- and intra-observer data recordings using the tapping process, wherein an ICC > 0.8 was considered to reflect good reliability.

## Results

### Reliability of the tapping process

To determine the reliability of the tapping process for data recording, three cases were randomly selected. The correlations detected using the frame-by-frame playback for the total number of EMs and EM bursts were 97.2% and 97.1%, respectively. The intra- and inter-observer reliability values for the total number of EMs and EM bursts were 0.99–0.98 and 0.99–0.96, respectively, which indicates a high level of reliability for data recording using the tapping process.

### Experimental outcomes and analyses

**Fig 1:**
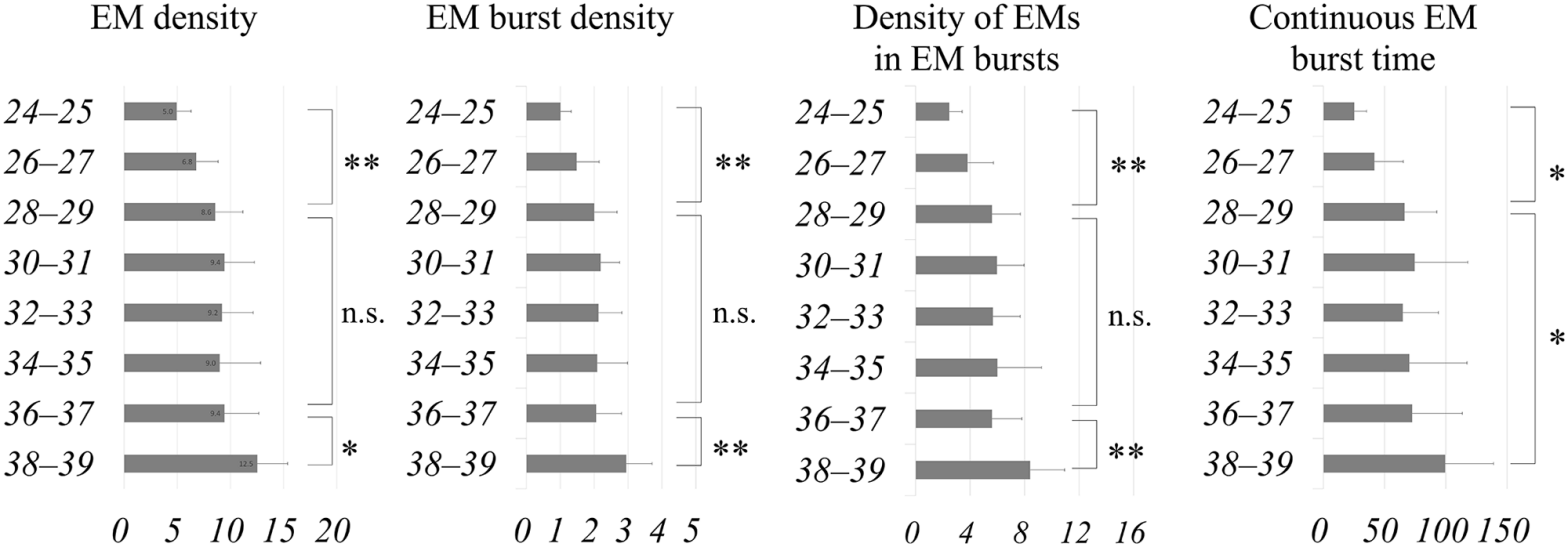
EM density, EM burst density, density of EMs in EM bursts, and continuous EM burst time. The vertical axis indicates the gestational weeks. The bars are represented as mean and standard deviation. *p < 0.05, **p < 0.01, n.s.; not significant.

Of the 86 cases, 84 featured an effective observation time of 80% or more. Further analyses were thus performed in these 84 cases. Table 2 shows the effective observation time, EM period rate (%), and NEM period rate (%) for each group. Fig 1. shows the measured EM variables. Table 3 shows the results obtained from a piecewise linear regression analysis of EM density, EM burst density, density of EMs in EM bursts and continuous EM burst time.

**Table 2:** Effective observation time and eye movement (EM)/non-eye movement (NEM) period rate.

Age group (weeks)	Effective observation time (min)	EM period rate (%)	NEM period rate (%)
24–25	55 ± 3	69 ± 8	31 ± 8
26–27	57 ± 3	68 ± 16	32 ± 16
28–29	57 ± 3	71 ± 9	29 ± 9
30–31	57 ± 3	74 ± 14	26 ± 14
32–33	58 ± 2	70 ± 7	30 ± 7
34–35	56 ± 4	70 ± 8	30 ± 8
36–37	57 ± 4	71 ± 15	29 ± 15
38–39	58 ± 3	66 ± 7	34 ± 7

Data are represented as mean ± standard deviation.

There were no significant differences between groups.

**Table 3:** Results obtained from piecewise linear regression analysis.

	*p* = 1		*p* = 2		*p* = 3	
	Critical points (weeks)	*Cp* value	Critical points (weeks)	*Cp* value	Critical points (weeks)	*Cp* value
EM density	(24–25)	4.8	(24–25), 28–29	4.2	(24–25), 28–29, 36–37*	0.47
EM burst density	(24–25)	8.4	(24–25), 28–29	6.2	(24–25), 28–29, 36–37*	0.48
Density of EMs in EM bursts	(24–25)	7.1	(24–25), 28–29	5.4	(24–25), 28–29, 36–37*	0.46
Continuous EM burst time	(24–25)	2.3	(24–25), 28–29*	1.6	(24–25), 28–29, 36–37	0.65

EM: eye movements; p: number of critical points; *Cp* value: Mallows' *Cp* value.

*The best combination of critical points selected according to *Cp* values.

**Fig 2:**
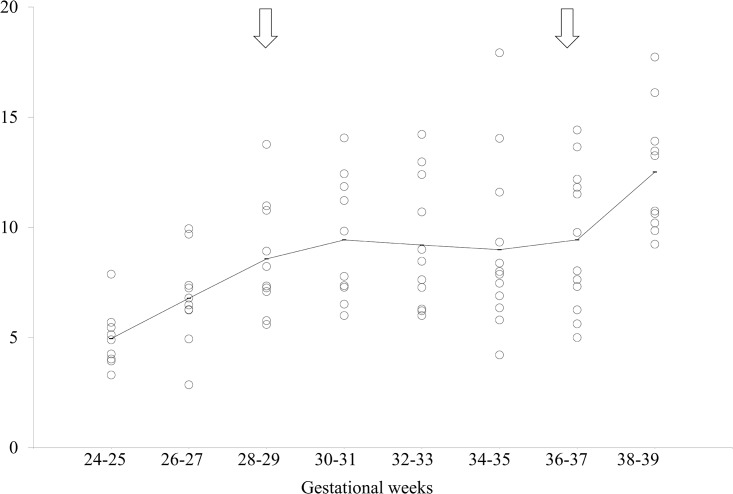
Scattergram of eye movement (EM) density. The horizontal axis indicates the gestational weeks and the vertical axis indicates the EM density. The solid line indicates the average of each group. Open arrows indicate the statistically defined critical points.

Two statistically significant critical points were found in EM density (Fig 2) at 28–29 weeks and 36–37 weeks of gestation (*Cp* = 0.47; Table 3), during which the mean EM densities were 8.6 and 9.4, respectively. EM density increased in the period from 24–25 weeks up to the first critical point at 28–29 weeks of gestation (*p <* 0.01). There was no significant difference between the first critical point (28–29 weeks) and the second critical point (36–37 weeks) (*p* = 0.48), between which the EM density remained rather constant. The EM density then increased from this second critical point to a time of 38–39 weeks (*p* = 0.01) (Fig 1).

**Fig 3:**
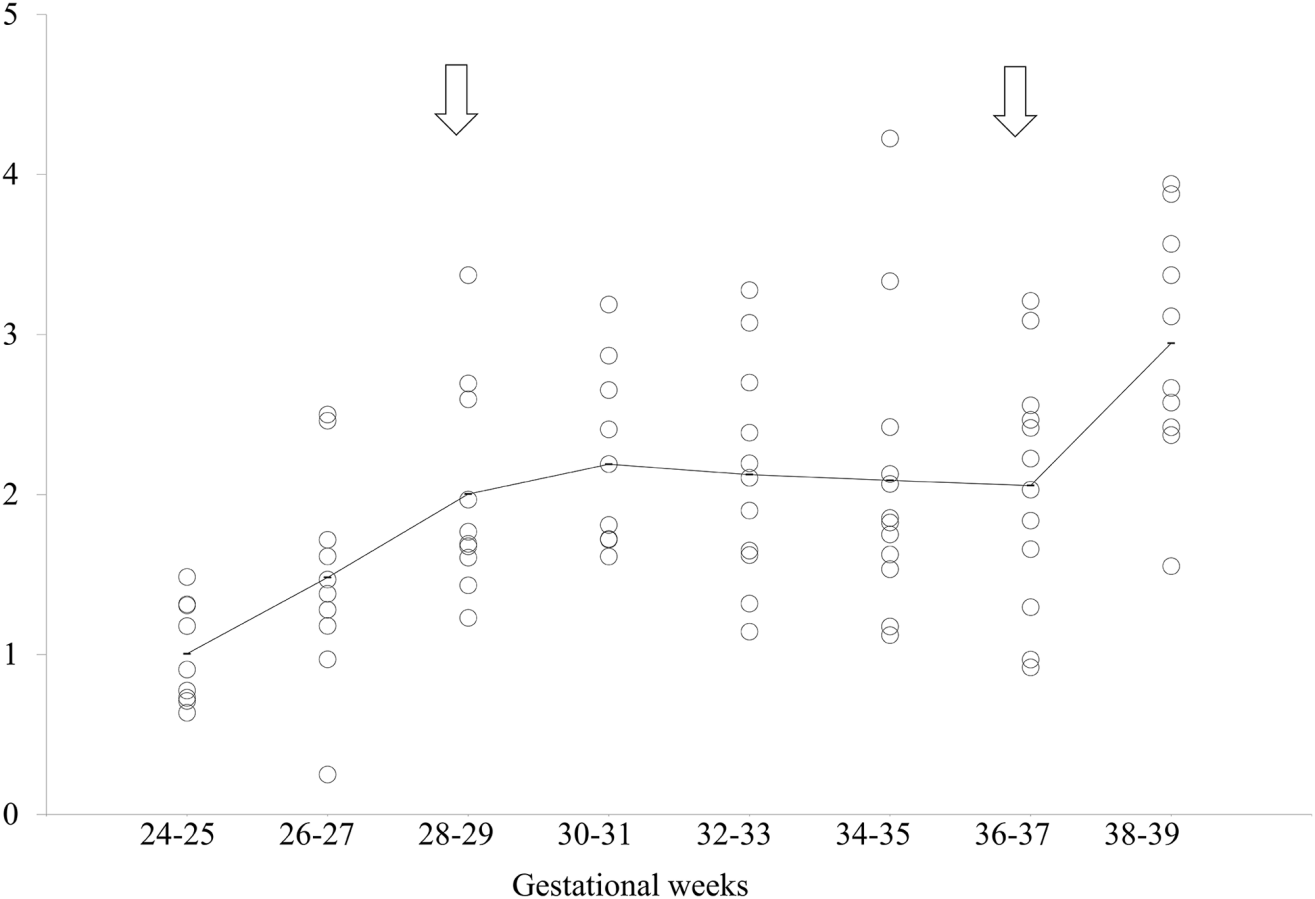
Scattergram of eye movement (EM) burst density. The horizontal axis indicates the gestational weeks and the vertical axis indicates the EM burst density. The solid line indicates the average of each group. Open arrows indicate the statistically defined critical points.

Two statistically significant critical points were found in the EM burst density (Fig 3) at 28–29 weeks and at 36–37 weeks of gestation (*Cp* = 0.48; Table 3), at which time the mean EM burst densities were 2.0 and 2.1, respectively. EM burst density increased from 24–25 weeks up to the first critical point at 28–29 weeks of gestation (*p* = 0.003). There was no significant difference between the first critical point (28–29 weeks) and the second critical point (36–37 weeks) (*p* = 0.86), between which the EM burst density remained rather constant. The EM burst density then increased in the period from this second critical point to 38–39 weeks (*p* = 0.004) (Fig 1).

**Fig 4:**
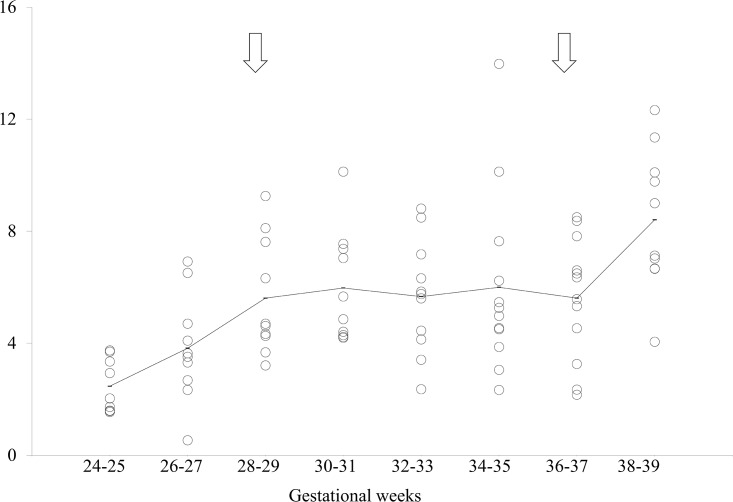
Scattergram of density of eye movements (EMs) in EM bursts. The horizontal axis indicates the gestational weeks and the vertical axis indicates the density of EMs in EM bursts. The solid line indicates the average of each group. Open arrows indicate the statistically defined critical points.

Two critical points were found in the density of EMs in EM bursts (Fig 4) at 28–29 weeks and at 36–37 weeks of gestation (*Cp* = 0.46; Table 3), during which the mean density of EMs in EM bursts was 5.6 and 5.6, respectively. The density of EMs in EM bursts increased from the period of 24–25 weeks up to the first critical point at 28–29 weeks of gestation (*p* = 0.003). There was no significant difference between the first critical point (28–29 weeks) and the second critical point (36–37 weeks) (*p* = 1.00), throughout which the density of EMs in EM bursts remained rather constant. It then increased from this second critical point to the time of 38–39 weeks (*p* = 0.005) (Fig 1).

**Fig 5:**
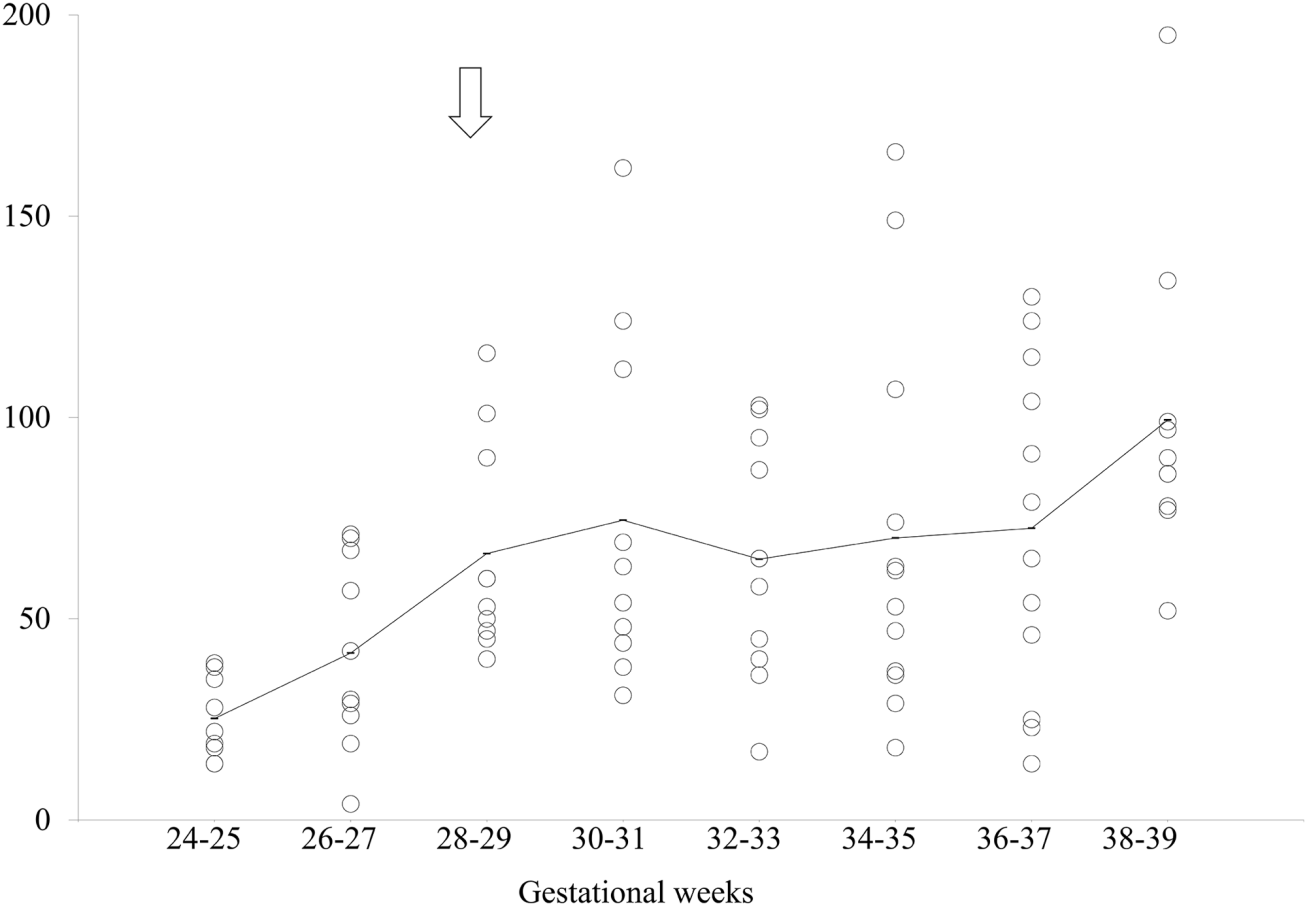
Scattergram of continuous eye movement (EM) burst time. The horizontal axis indicates the gestational weeks and the vertical axis indicates the continuous EM burst time. The solid line indicates the average of each group. Open arrows indicate the statistically defined critical points.

One critical point was found in the continuous EM burst time (Fig 5) at 28–29 weeks of gestation (*Cp* = 1.6; Table 3), at which time the mean EM burst time was 65 sec. The continuous EM burst time increased from the period of 24–25 weeks up to the first critical point at 28–29 weeks of gestation (*p* = 0.013). Afterwards, there was a tendency to increase from this first critical point to 38–39 weeks (*p* = 0.038) (Fig 1).

## Discussion

### Main findings

In the present study, two critical points at 28–29 and 36–37 weeks of gestation were identified for the EM density, EM burst density, and density of EMs in EM bursts. The EM density, EM burst density and density of EMs in EM bursts had the same critical points, which suggests that the development of EM density is associated with the development of the EM burst function.

### Comparison with existing literature

In a previous study, Inoue et al. classified the number of EMs/min into low, moderate, and high frequency EM categories, and clarified their mode of occurrence with regard to advancing gestation [17]. The authors reported that moderate-frequency EMs began to increase between 22 and 25 weeks, while high frequency EMs increased between 30 and 33 weeks of gestation. Moreover, Inoue et al. reported that these low, moderate, and high frequency EM classifications corresponded to the type I, type II-III, and type IV EM categories proposed by Birnholz et al., respectively [16]. In the study by Birnholz et al., the frequency of the type III category was the highest between weeks 29–31 of gestation [16]. The present results indicate that the increase in high frequency and type III categories might be due to the observed increase in EM bursts.

In infant studies, EM bursts increase until 2 months and then plateau at 4 months of age [25]. In other words, the frequency of EM bursts changes according to the developmental period. In a study of preterm birth infants, REM sleep activity correlated with cognitive development at six months of age [14]. In contrast, Becker et al. used REM storms instead of EM bursts to examine infants at six months of age, wherein the frequency of the "REM storms" was used as an indicator of central nervous system development. In addition, Becker et al. reported that at six months, a high frequency of REM storms was correlated with a delay in mental development at 1 year of age [26]. This and similar studies indicate that the REM activity at each period of development is related to neurodevelopmental prognosis. Moreover, these studies suggest that optimum REM activity is extremely important for the normal progression of each developmental period.

The vestibular nucleus has been linked to the production of EM bursts, wherein the blockage of this nucleus in cats is associated with the selective blockade of EM bursts [27]. Similarly, studies of patients with severe cases of Wernicke-Korsakoff syndrome, who typically present with a destruction of the vestibular nucleus, reported the absence of REM burst activity [28]. The vestibular nucleus can be cytoarchitectonically divided into two nuclei (lateral and medial) from 21 weeks of gestation [29]. Although there have been no reports between this period of development and birth, at 2 months after birth, growth-associated protein 43, which is commonly expressed in nerve cells during development, is no longer present in the vestibular nucleus [30]. Growth-associated protein 43 marks active synaptogenesis; thus, its absence by 2 months of age suggests that this nucleus has achieved synaptic maturity by this time. This observation is consistent with previous studies, wherein EM bursts increased until approximately 2 months after birth [25]. In the present study, EM burst density was significantly increased up to 28–29 weeks of gestation, which is a period that correlates with the maturity of the vestibular nuclei. In studies of preterm birth infants, at approximately 28 to 30 weeks of gestation, body movement, and EM expression cycles become synchronized during sleep [31]. The period in which body movement and EM synchrony arises is believed to be a precursor of REM sleep, and is often referred to as “active sleep” [1]. During this time, the brain centers for REM sleep begin to function. As mentioned above, 28–29 weeks of gestation appears to represent the period at which the brain centers for REM sleep and EM bursting begin to function. After the critical point of 36–37 weeks of gestation, the present study identified a significant increase in EM activity. The EEG-based study of human preterm infants has suggested that after 36 weeks of gestation, EEG responses to sensory stimulation closely resembled a more mature pattern indicative of cortical maturation [32]. Although the change was sensory system-specific, it was related to the establishment of distinct sleep states in infants [32,33]. Thus, it appears that cortical development is accompanied by the changes occurring in late gestation.

### Strengths and limitations

The present study is the most detailed study to date of EM activity in human fetuses.

One limitation of the present study relates to the limited time period (i.e., within gestation) studied; we could not compare the data to subsequent neurological prognoses. Moreover, future research should use a larger number of cases. Nonetheless, consistent tendencies were identified that were indicative of a developmental process of EM activity.

## Conclusions

The EM activity of human fetuses increased up to 28–29 weeks of gestation, and further increased from 36–37 to 38–39 weeks of gestation. These results could be used to foster the development of an index of fetal neurological function, allowing for correlation with studies of fetuses and infants with abnormal brain structures and neurodevelopmental outcomes, as well as to provide valuable normative data to eventually allow for neurological prognosis and prediction in utero.

## Supporting information

S1 Table Individual data depicted in Fig 1. (XLSX)

S2 Table Individual data depicted in Fig 2. (XLSX)

S3 Table Individual data depicted in Fig 3. (XLSX)

S4 Table Individual data depicted in Fig 4. (XLSX)

S5 Table Individual data depicted in Fig 5. (XLSX)
